# The prevalence and related factors of metabolic syndrome in outpatients with first-episode drug-naive major depression comorbid with anxiety

**DOI:** 10.1038/s41598-021-81653-2

**Published:** 2021-02-08

**Authors:** Yinghua Zhong, Manji Hu, Qiang Wang, Zhendong Yang, Na Zhu, Fei Wang, Xiyan Zhang, Chengfang Zhang, Jie Min, Hao Wang, Fazhan Chen, Xudong Zhao, Xiangyang Zhang

**Affiliations:** 1 Shanghai Pudong New Area Mental Health Center, Tongji University School of Medicine, Shanghai, China; 2 Division of Medical Humanities and Behavioral Sciences, Tongji University School of Medicine, 1239 Siping Road, Postbox No. 244, Shanghai, 200092 China; 3 CAS Key Laboratory of Mental Health, Institute of Psychology, Chinese Academy of Sciences, Beijing, China; 4 Department of Psychology, University of Chinese Academy of Sciences, 16 Lincui Road, Chaoyang, Beijing, 100101 China

**Keywords:** Psychiatric disorders, Anxiety, Depression

## Abstract

Metabolic syndrome (MetS) is associated with depression, but its role in major depressive disorder comorbid with anxiety (AMD) is unclear. This study aimed to investigate the prevalence and clinical correlates of MetS in first-episode drug-naive (FEDN) patients with AMD in a Chinese Han population. In total, 1380 FEDN outpatients with AMD were recruited in this cross-sectional study. The sociodemographic features, clinical characteristics, history of suicide attempts, thyroid-stimulating hormone (TSH) levels, and MetS parameters of each subject were evaluated. All subjects were rated on the Hamilton Depression Rating Scale (HAM-D), Hamilton Anxiety Rating Scale (HAM-A), and the Positive and Negative Syndrome Scale positive symptom subscale. The prevalence of MetS among AMD patients was 8.04%. Compared to the non-MetS group, age, age of onset, TSH level, HAM-A and HAM-D scores, history of attempted suicide, and comorbid psychiatric symptoms were higher in the MetS group. Those in this group were also more likely to be married, and they had a lower educational level. Furthermore, age, psychiatric symptoms, suicide attempts, and higher TSH levels were independently associated with MetS in AMD patients. This study suggests a lower prevalence of MetS in FEDN patients with AMD in a Chinese Han population. Older age, comorbid psychiatric symptoms, history of attempted suicide, and higher TSH levels are related factors for MetS in AMD patients.

## Introduction

Metabolic syndrome (MetS) is an important global public health problem and comprises a group of complex risk factors, including obesity, dyslipidemia, hyperglycemia, and hypertension^1^. The current consensus is that MetS may lead to type 2 diabetes and cardiovascular disease (CVD) and seriously reduce the individual’s quality of life^2,3^. Data from different countries and races show that the overall pooled prevalence of MetS ranges from 3.6% in Asia to 78.2% in Europe^4–9^. Accumulating evidence shows that MetS may be related to depression and its severity^10–13^. An epidemiological survey of middle-aged and older individuals shows that major depressive disorder (MDD) increases the risk of MetS by nearly four times^10^; however, the mechanism by which depression affects MetS is unclear. Previous studies have shown that this effect may be related to immune inflammation activation, oxidative and nitrosative stress pathways, HPA axis dysfunction, gene mutations, unhealthy lifestyles, and other biological disorders^14–17^. The role of anxiety in MetS has been gradually recognized in recent years. However, the results of studies on the association between anxiety and MetS are inconsistent. Some authors have reported that anxiety, rather than depression, is associated with MetS^18,19^. Anxiety severity has a greater impact on MetS than depression severity^20^. However, other studies have failed to replicate this association^10,21^. Interestingly, in a recent meta-analysis, cross-sectional studies showed a significant positive correlation between anxiety and MetS, but only two cohort studies found that the association between anxiety and MetS was almost negligible^22^.

Anxiety and depression often coexist, indicating a more severe course and outcome. A recent study reported that these two factors lead to a higher prevalence of diabetes by affecting common behavioral and metabolic factors^3^. Another study found that depression and anxiety increased the mortality rates of patients with oral anticoagulation by 22% and 11%, respectively^23^.

The relationship between AMD and MetS has been examined; however, the results of these studies are contradictory. For example, one study reported that MDD patients with anxiety were more closely associated with MetS than MDD patients without anxiety^24^, but another cross-sectional study reached a different conclusion^25^. A meta-analysis by Bystritsky et al. found that the relationship between medical diseases and mental diseases is very complicated. Diseases may cause biological changes in the brain, which may induce or strengthen psychopathology. Mental illness syndrome, in turn, may cause behavioral deterioration or change the course of the medical disease. This effect may be a two-way relationship^26^. For example, an animal study showed that a diet rich in saturated fatty acids and fructose caused metabolic disturbances, triggering anxiety and depression-like behaviors in rats^27^. Other studies found that anxiety, depression, suicide attempts, and elevated thyrotropin caused obesity, increased blood lipids, increased blood glucose, higher body mass index (BMI), elevated blood pressure and other metabolic abnormalities^28–30^. Unfortunately, the relationship between these MetS risk factors and patients with depression and anxiety has not been systematically reported. Many studies have reported the characteristics of MetS in MDD patients; however, few studies have explored the specific characteristics of MetS in MDD patients with coexisting anxiety. Currently, there is no large-scale study on the relationship between AMD and MetS in Han Chinese patients. Therefore, this study aimed to identify the prevalence and related factors of MetS in first-episode drug-naive (FEDN) Chinese Han outpatients with AMD through a cross-sectional design.

## Methods

### Subjects and settings

In this cross-sectional study, all AMD patients were recruited from the outpatient psychiatric department of a general hospital in Taiyuan, Shanxi Province, China, from 2015 to 2017. All patients met the following recruitment criteria: (1) aged 18–60 years and Han nationality; (2) had never received antidepressants or other psychotropic drugs; (3) met the criteria for MDD and had been diagnosed by two experienced psychiatrists independently according to the Chinese version of the Structured Clinical Interview for the DSM-IV; (4) had depressive symptoms, with a 17-item Hamilton Depression Rating Scale (HAM-D) score ≥ 24; and (5) had anxiety symptoms, with a 14-item Hamilton Anxiety Rating Scale (HAM-A) score ≥ 18. The exclusion criteria were (1) serious physical diseases; (2) substance abuse in the past 6 months; (3) severe personality disorder; and (4) current pregnancy or lactation.

All enrolled patients provided written informed consent after a full explanation of the study. The research plan was approved by the institutional review board (IRB) of the First Medical College of Shanxi Medical University and conducted according to the Declaration of Helsinki.

### Clinical measurements

The sociodemographic and clinical data of the participants were collected including sex, age, age of onset, marital status, educational level, disease course, BMI, blood pressure, suicidal behavior, and biological indicators by laboratory testing.

The Positive and Negative Syndrome Scale (PANSS) positive symptom subscale was used to assess psychiatric symptoms. The scale includes seven items with a total score ranging from 7 to 49. Consistent with previous work, a score ≥ 15 was defined as having psychiatric symptoms^31,32^.

Depressive symptoms were evaluated by HAM-D, with 17 items and five factors. The scale includes eight items rated on a five-point scale ranging from 0 to 4 and nine items rated on a three-point scale ranging from 0 to 2. Total scores of < 8, 8–17, and ≥ 24 are rated as no depression, mild to moderate depressive symptoms and severe depression, respectively^33^.

We employed the 14-item HAM-A to evaluate anxiety symptom severity. Total scores of 18–24 and 25–30 were rated as mild to moderate and moderate to severe anxiety, respectively^34^. This study used 18 points as a cutoff to divide participants into two groups: with or without anxiety symptoms.

A destructive act with which an individual intended to end his or her life but that did not lead to death was defined as a suicide attempt. The detailed investigation method has been described previously^35^.

To ensure the reliability and consistency of the scores of these scales during the study period, two psychiatrists who participated in the evaluation received training in the application of HAM-D, HAM-A and PANSS before the study. After training, the correlation coefficient of positive symptom scores of HAM-D, HAM-A and PANSS was maintained at ≥ 0.8. The assessors were blinded to the clinical condition of patients.

### Biomarker measurements

To measure parameters including thyroid-stimulating hormone (TSH), triglycerides, high-density lipoprotein cholesterol (HDL-C), and blood glucose levels, fasting venous blood samples of all participants were collected between 6 and 8 a.m. Simultaneously, the patient's BMI, systolic blood pressure (SBP) and diastolic blood pressure (DBP) were measured. All blood samples were transported promptly to the laboratory center of the First Clinical Medical College of Shanxi Medical University and tested before 11 a.m. on the same day.

The diagnostic criteria for MetS were based on the diagnostic criteria of the Diabetes Branch of the Chinese Medical Association^36^, which conformed to at least three of the following four items: (1) BMI ≥ 25 kg/m^2^; (2) fasting plasma glucose (FPG) ≥ 6.1 mmol/L and/or 2-h postprandial glucose ≥ 7.8 mmol/L and a diagnosis of type 2 diabetes mellitus (T2DM); (3) SBP/DBP ≥ 140/90 mmHg and/or diagnosis of hypertension; and (4) fasting serum triglycerides ≥ 1.7 mmol/L and/or fasting HDL-C concentration < 0.9 mmol/L for males or < 1.0 mmol/L for females.

### Statistical analysis

All statistical analyses were carried out with SPSS 20.0. The prevalence of MetS among AMD patients was expressed as the number of cases (percentage). Student’s t test for continuous variables and chi-square tests for categorical variables were used for comparisons of related variables among people with different characteristics.The difference in educational level between MetS and non-MetS among AMD patients was evaluated with a nonparametric test. The influencing factors of MetS were analyzed by single-factor and multifactor binary logistic regression. A value of P < 0.05 was regarded as statistically significant.

## Results

### Demographic and clinical characteristics of FEDN patients with AMD

Among the 1380 AMD patients, 905 were female (65.6%), and 475 were male (34.4%). The average age of the patients was 34.96 ± 12.477 years, ranging from 18 to 60 years. The average age of onset was 34.75 ± 12.355 years, ranging from 17 to 60 years. The average duration of illness was 6.32 ± 4.683 months, ranging from 1 to 28 months. The educational levels were as follows: junior high school, 332 cases (24.1%); high school, 602 cases (43.6%); university, 371 cases (26.9%); and post-graduate, 75 cases (5.4%). In total, 981 (71.1%) were married, and 399 (28.9%) were not married.

### Differences in clinical characteristics between MetS and non-MetS among AMD patients

Among the AMD patients, there were 111 cases of MetS (8.04%) and 1269 cases without MetS (91.96%).

As shown in Table 1, among AMD patients, the HAM-A scores, HAM-D scores, systolic pressure, diastolic pressure, BMI, TSH, blood glucose, triglycerides, and HDL-C serum levels in the MetS group were significantly higher than those in the non-MetS group (all P < 0.05). Additionally, age, age at onset, marital status, educational level, suicide attempts, and psychiatric symptoms were significantly different between the two groups (all P < 0.05). However, there were no significant differences in illness duration or sex (both P > 0.05).

**Table 1:** Sociodemographic and clinical characteristics of AMD patients with and without MetS.

	Total patients (n = 1380) Mean ± SD	Patients without MetS (n = 1269) Mean ± SD	Patients with MetS (n = 111) Mean ± SD	*t*	*P* value
Age (years)	34.96 ± 12.477	34.41 ± 12.366	41.14 ± 12.120	− 5.507	< 0.001
Age at onset (years)	34.75 ± 12.355	34.22 ± 12.238	40.88 ± 12.075	− 5.510	< 0.001
Illness duration (months)	6.32 ± 4.683	6.26 ± 4.648	6.96 ± 5.050	− 1.499	0.134
BMI (kg/m^2^)	24.367 ± 1.933	24.247 ± 1.923	25.738 ± 1.469	− 9.966	< 0.001
HAM-D	30.84 ± 2.814	30.77 ± 2.827	31.68 ± 2.538	− 3.271	0.001
HAM-A	21.90 ± 2.951	21.80 ± 2.898	23.02 ± 3.317	− 3.749	< 0.001
TSH (uIU/L)	5.285 ± 2.687	5.093 ± 2.590	7.483 ± 2.812	− 9.256	< 0.001
Blood glucose (mmol/L)	5.425 ± 0.663	5.344 ± 0.605	6.355 ± 0.590	− 17.288	< 0.001
Triglycerides (mmol/L)	2.195 ± 0.985	2.140 ± 0.962	2.823 ± 1.029	− 7.128	< 0.001
HDL-C (mmol/L)	1.214 ± 0.294	1.227 ± 0.292	1.067 ± 0.272	5.574	< 0.001
Systolic pressure (mmHg)	120.10 ± 11.032	119.28 ± 10.682	129.53 ± 10.616	− 9.706	< 0.001
Diastolic pressure (mmHg)	76.37 ± 6.813	75.89 ± 6.460	81.81 ± 8.269	− 7.345	< 0.001

Characteristic	Total patients (n = 1380) n (%)	Patients without MetS (n = 1269) n(%)	n (%Patients with MetS (n = 111) n (%)	χ^2^/Z	*P* value
**Sex **					
Male	475 (34.4)	440 (34.7)	35 (31.5)	0.446	0.534
Female	905 (65.6)	829 (65.3)	76 (68.5)		
**Marital status**					
Not married	399 (28.9)	380 (29.9)	19 (17.1)	8.172	0.004
Married	981 (71.1)	889 (70.1)	92 (82.9)		
**Psychiatric symptoms**					
No	1210 (87.7)	1128(88.9)	82 (73.9)	21.305	< 0.001
Yes	170 (12.3)	141 (11.1)	29 (26.1)		
**Suicide attempts**					
No	1045 (75.7)	987(77.8)	58 (52.3)	36.179	< 0.001
Yes	335 (24.3)	282(22.2)	53 (47.7)		
**Educational level**					
Junior high school	332 (24.1)	293(23.1)	39 (35.1)	6.408	0.011
High school	602 (43.6)	558 (44.0)	44 (39.6)		
University	371 (26.9)	349 (27.5)	22 (19.8)		
Post-graduate	75 (5.4)	69 (5.4)	6 (5.4)		

*BMI* body mass index, *HAM-D* Hamilton depression rating scale, *HAM-A* Hamilton anxiety rating scale, *TSH* thyrotropin, thyroid-stimulating hormone, *HDL-C* high-density lipoprotein cholesterol.

### Risk factors for MetS in AMD patients

#### Risk factors related to MetS by univariate binary logistic analysis

To carry out the stepwise binary logistic regression analysis of variables, qualitative and quantitative variables were graded and assigned, as detailed in Table 2.

**Table 2:** Description table of hierarchical assignment of included influencing factors.

Variables	Hierarchical assignment
**Sex**	
Female	0
Male	1
**Marital status**	
Not married	0
Married	1
**Suicide attempts**	
No	0
Yes	1
**Psychiatric symptoms**	
No	0
Yes	1
**TSH levels**	
Normal	0
Higher	1
**MetS**	
No	0
Yes	1
**Age group (years)**	
< 20	1
20–29	2
30–39	3
≥ 40	4
**HAM-A score**	
< 18	1
18–24	2
≥ 25	3
**HAM-D score**	
< 31	1
31–38	2
≥ 39	3

In the univariate binary logistic analysis, MetS was a dependent variable and actual age, sex, illness duration, marital status, suicide attempts, and psychiatric symptoms were covariates. The results in Table 3 shows that age, TSH levels, psychiatric symptoms, suicide attempts, marital status, HAM-A score, and HAM-D score were independently associated with MetS of AMD.

**Table 3:** Risk factors related to MetS by univariate binary logistic analysis.

Covariate	Coefficients *B*	Std.error	Wals value	*P*	OR (95%CI)
Age	0.525	0.109	23.238	< 0.001	1.690 (1.365–2.092)
Sex	− 2.496	0.112	495.191	< 0.001	0.082 (0.512–1.153)
Marital status	0.781	0.251	9.695	0.002	2.183 (1.335–3.569)
Suicide attempts	1.244	0.194	40.977	< 0.001	3.469 (2.370–5.077)
Psychiatric symptoms	1.172	0.230	25.896	< 0.001	3.230 (2.056–5.073)
HAM-D score	0.143	0.032	20.073	< 0.001	1.154 (1.084–1.228)
HAM-A score	0.134	0.025	27.825	< 0.001	1.143 (1.088–1.201)
TSH levels	1.453	0.267	29.724	< 0.001	4.276 (2.536–7.209)

#### Risk factors related to MetS by multivariate binary logistic analysis

Multivariate binary logistic analysis was performed to analyze the risk factors for MetS among AMD patients. MetS was the dependent variable, and covariates included marital status, presence of suicide attempts, presence of psychiatric symptoms, TSH levels, HAM-D score, HAM-A score, and age group. In the stepwise binary logistic analysis equation, risk factors for MetS included age, TSH levels, psychiatric symptoms, and suicide attempts. Table 4 shows the results of the multivariate logistic models.

**Table 4:** Risk factors related to MetS by multivariate binary logistic analysis.

Covariate	Coefficients *B*	Std.error	Wals value	*P*	OR (95%CI)
Age	0.474	0.143	11.006	0.001	1.606 (1.214–2.124)
TSH levels	1.176	0.272	18.676	< 0.001	3.242 (1.902–5.526)
Suicide attempts	0.840	0.214	15.370	< 0.001	2.317 (1.522–3.527)
Psychiatric symptoms	0.547	0.255	4.607	0.032	1.728 (1.049–2.848)

## Discussion

To the best of our knowledge, this is the first observational study to explore the prevalence and clinical correlates of MetS in FEDN patients with AMD in a Chinese population. We found that the proportion of MetS among AMD cases was 8.04%. Our study revealed that MetS was associated with older age, being married, history of suicide attempts, comorbid psychiatric symptoms, higher blood serum levels of TSH, a higher HAM-A score, and a higher HAM-D score, indicating that they may be promising factors for the evaluation of MetS in AMD patients. Moreover, after controlling for demographics, we found that anxiety and depressive symptoms, older age, history of suicide attempts, comorbid psychiatric symptoms, and higher blood serum levels of TSH remained independent risk factors for MetS. This implies that the abovementioned features may increase the risk of MetS in AMD patients.

Remarkably, there is currently little research on the prevalence of MetS among AMD patients. Several previous studies have reported that the prevalence rate of MetS among MDD patients is 26.2–43%^10,13,37^, which is higher than that among healthy controls. Additionally, a systematic review and meta-analysis showed that the combined prevalence of MetS among MDD patients was 31.3%^38^. In contrast to previous studies, our cross-sectional study showed that the prevalence of MetS among AMD outpatients was lower than that of age-standardized MetS in the Chinese Han population (8.04% vs 19.8%)^6^. Akbari et al. found that the prevalence of MetS among patients with anxiety was lower than that among healthy controls^39^, which was consistent with our results. The prevalence of MetS varies dramatically due to the following factors. First, the participants in this study were MDD patients comorbid with anxiety. According to the DSM-IV diagnostic criteria, patients may have a poor appetite, resulting in significant weight loss. A Japanese study also found that hyperphagia in patients with atypical depression may be a very important mediator that is positively related to MetS^40^. Second, a previous study suggests that antidepressants may be a risk factor for MetS^11^. Goldbacher et al. found that a history of recurrent depression rather than a single depressive episode increased the risk of developing MetS^41^ . However, the subjects included in this study were first-episode and drug-naive patients, which may also be the reason for inconsistency with other studies. Third, the use of various tools to assess emotional status or different MetS diagnostic criteria may also affect the results.

In the univariate binary logistic analysis, we found that the HAM-A score and HAM-D score may be risk factors for MetS in AMD patients. However, after controlling for age and marital status, we found that they were not independent risk factors for MetS in AMD patients. This is inconsistent with previous studies, including several that have found that anxiety is a risk factor for MetS^18,42^. Other studies have also found that depression and anxiety further increase the risk of T2DM and cardiovascular events^43,44^. Mattei et al. found that MetS is not related to depression or anxiety but is related to anxiety comorbid with depression^24^. However, some authors have different viewpoints. In a meta-analysis that examined data from cross-sectional and longitudinal cohort studies, cross-sectional studies showed that anxiety was significantly positively correlated with MetS, but the cohort study suggested that the relationship between anxiety and MetS was negligible^22^. One prospective cohort study found that the prevalence of MetS among anxiety patients was lower than that among healthy subjects and that anxiety symptoms were negatively correlated with MetS^39^. Therefore, the causal relationship between anxiety and metabolism should be explored in longitudinal cohort studies with larger sample sizes, and further studies related to mechanisms in animal model studies may obtain more reliable evidence.

Although this study did not identify anxiety as an independent risk factor for MetS in patients with AMD, we found that in AMD patients, the groups with and without MetS had different demographic and clinical characteristics. In addition to disease course and sex, other factors including age, age at onset, BMI, HAM-D score, HAM-A score, TSH levels, blood glucose, triglycerides, blood pressure, HDL-C levels, married, psychosis symptoms, suicide attempts, and education level showed significant differences between the two groups. Some of these factors, including high blood glucose, high triglycerides, low HDL-C, high BMI, and high blood pressure, have been recognized as risk factors for MetS^1,45^. Additionally, previous studies have found that the HAM-A and HAM-D scores of MetS patients are higher than those of non-MetS patients^20,46^, which is consistent with our findings, indicating that the relationship between anxiety, depression, and metabolism is complex. The effect of anxiety on metabolism is mediated by comorbid depression, which may be a reasonable explanation^47^.

Furthermore, Akbari et al. found that older women had a higher prevalence of MetS^39^, but we found that although the proportion of women in the MetS group (68.5%) was higher than that in the non-MetS group (65.3%), no significant difference was found. A longitudinal 6-year follow-up study found that sex was not related to MetS^48^. A recent large-scale cross-sectional survey of 98,042 people in China found no significant difference in the prevalence of MetS between men and women, which also supports our results^49^. Additionally, we found that older age is a risk factor for MetS, which is in line with previous findings^6,49,50^. Therefore, we speculate that the following reasons may lead to sex differences in the distribution of MetS. First, changes in hormone levels in postmenopausal women may affect metabolism. A recent study showed that women over the age of 50 years who have insomnia are at greater risk of developing MetS. However, this is not obvious among women under the age of 50 years^51^. The average age of the participants in this study was younger than that of the above study and may be a protective factor. Second, men generally have a higher smoking rate than women, and tobacco use disorders in men may lead to blood lipid profiles and other related risk pathways, which may increase the risk of metabolic disorders in men^52^.

Regarding psychotic symptoms, previous studies have demonstrated the potential interactions between psychotic symptoms and MetS^18,53^. Our study also found that compared with those with no psychiatric symptoms, AMD patients with psychiatric symptoms had a nearly twofold increased risk of MetS (OR 1.728, 95% CI 1.049–2.848), indicating that psychiatric symptoms may further increase the risk of MetS in AMD patients. Additionally, the DSM-5 diagnostic criteria indicate that MDD with psychotic features is a serious subtype of MDD. Its presence makes treatment more difficult and often predicts worse treatment outcomes. We suggest the following explanations. First, psychiatric symptoms such as hallucinations and delusions may affect the patient’s judgment and self-management ability, resulting in poor compliance with the treatment of physical diseases and missed opportunities for intervention. Second, patients with psychotic disorders often have poor lifestyle habits, such as high smoking rates, obesity, lack of exercise, and malnutrition and are more prone to metabolic disorders^52^. Third, to control the psychiatric symptoms of MDD patients, it is necessary to combine antipsychotic drugs with antidepressants in clinical treatment, but some studies have found that these drugs may also be risk factors for MetS^11,54^. Fourth, abnormalities in disorders of immune metabolism and the endocrine balance system may be the pathophysiological mechanism for comorbidities of severe psychotic disorders and MetS^55^. AMD accompanied by mental disorders may enhance this effect. Therefore, we should pay attention to the psychotic symptoms of patients with depression. If antipsychotic drugs must be used in combination, drugs with fewer metabolic effects should be selected.

In recent years, the relationship between thyroid hormone and MetS has been a focus of research. Many studies have found that a high TSH level within the normal range is related to MetS^28,56,57,58^. A study by Ren et al. found that MetS components, such as BMI, body weight, blood pressure, and blood lipids, are affected by TSH levels^59^. A large-scale population-based study with a 7-year follow-up showed that continuous TSH levels and MetS prevalence were positively correlated^60^, indicating that there may be a causal relationship between TSH levels and MetS prevalence. Interestingly, our study also found that higher TSH levels tripled the risk of MetS in AMD patients (OR 3.242, 95% CI 1.902–5.526). Although the specific mechanism remains unclear, some authors have reported that elevated TSH levels may lead to endothelial dysfunction and insulin resistance, which may lead to MetS and related comorbidities^28,61,62^.

Our study found that attempted suicide increased the risk of MetS in AMD by more than double (OR 2.317, 95% CI 1.522–3.527). However, another study found no difference in MetS or blood lipid levels between suicidal patients and nonsuicidal patients^63^. However, the study sample comprised hospitalized patients with bipolar disorder who were receiving drug treatment. Additionally, the sample size was only 50 cases, which may lead to a type II error. Therefore, compared with those of our study (including 1380 patients), the findings of the above study are less convincing. Interestingly, a previous longitudinal study by Goldman-Mellor et al. supported our conclusion^64^. They conducted a long-term follow-up study of 1037 complete birth cohorts, 95.4% of whom were followed up to 38 years of age, and finally found that a history of suicide attempts during youth may predict future MetS. There are several possible reasons. First, suicide may be associated with mental health problems. Specifically, suicidal behavior in depression is a sign of serious illness. Second, people who attempt suicide may have more problems with substance abuse and impulsive violence than those who do not. Furthermore, these behaviors may be accompanied by more physical health problems, which in turn exacerbate suicide attempts. Taken together, our data show that among patients with AMD, a history of suicide attempts may indicate a greater risk of MetS and that appropriate intervention is needed to strengthen the secondary prevention of MetS in AMD patients.

Several limitations of this study should be noted. First, our findings should be considered preliminary and should be confirmed by other studies before we can draw clear conclusions. Second, the most commonly used diagnostic criteria for MetS worldwide are the International Diabetes Federation (IDF) and the National Cholesterol Education Adult Treatment Panel III-R (NCEP ATP III). However, the diagnostic criteria for MetS in this study were determined by the Diabetes Branch of the Chinese Medical Association based on the study of MetS in China, which is more consistent with the actual Chinese characteristics. Therefore, according to this standard, we did not collect waist circumference data. Although an international Joint Scientific Statement emphasizes that the measurement of waist circumference should vary by ethnicity and that abdominal obesity should not be used as a prerequisite for diagnosis, it is believed that waist circumference will still be a useful screening tool^1^. Third, the cross-sectional design of our study cannot explain the causal relationship between MetS and risk factors in AMD patients, so this relationship should be confirmed by prospective cohort studies. Fourth, our sample comprises outpatients. Therefore, the findings of this study cannot be extended to patients in other environments, such as inpatients or community patients. Fifth, data on relatively few variables were collected in this study. We did not collect data on other features related to MetS, such as lifestyle habits, substance abuse, comorbidities, personality traits, and family history of anxiety and MetS. Finally, although there are advantages in the study of FEDN patients in this study, there may also be disadvantages. Deficiencies may be present because depression may first occur in patients with unipolar depression or in patients with bipolar disorder. Although we made the second diagnosis during the 3–6 months of follow-up and included only patients who were also diagnosed with MDD at both time points, we cannot rule out the possibility that some diagnoses may translate into bipolar disorder.

In conclusion, our results showed that the prevalence of MetS in FEDN Chinese Han outpatients with AMD was 8.04%. Compared with the non-MetS group, the MetS group was older, had an older age of onset, had a higher serum TSH level, had higher HAM-A and HAM-D scores, had a lower educational level, was more likely to be married, was more likely to attempt suicide and had combined psychiatric symptoms. Moreover, older age, psychiatric symptoms, suicide attempts, and higher TSH levels were independently associated with MetS in AMD patients. However, the HAM-A and HAM-D scores were not independent risk factors for MetS in patients with AMD. Additionally, due to the study limitations, such as its cross-sectional design and lack of some metabolic data, the findings of this study should be considered preliminary and confirmed in future studies using a longitudinal design.
